# Preimplantation sex selection via in vitro fertilization: time for a reappraisal

**DOI:** 10.1016/j.xfre.2023.05.006

**Published:** 2023-05-26

**Authors:** Vitaly A. Kushnir, Eli Y. Adashi, I. Glenn Cohen

**Affiliations:** a Department of Obstetrics and Gynecology, University of California Irvine, Orange, California; b Division of Medicine and Biological Sciences, Brown University, Providence, Rhode Island; c Petrie-Flom Center for Health Law Policy, Biotechnology, and Bioethics, Harvard University, Cambridge, Massachusetts

**Keywords:** In vitro fertilization, preimplantation genetic testing, sex selection

## Abstract

In recent years, there has been rapid increase in the availability of elective sex selection via genetic testing of preimplantation embryos created through in vitro fertilization. We explore the standing of this ethically controversial practice in the context of a changing legal landscape after the Dobbs v Jackson Women’s Health decision by the US Supreme Court.

The use of in vitro fertilization (IVF) continues to increase. In vitro fertilization currently accounts for the conception of 2%–5% of all newborns in many developed nations (1). Moreover, IVF is increasingly coupled with sophisticated genetic testing of embryos. In the United States, the proportion of IVF cycles that use preimplantation genetic testing (PGT) increased from 4.5% in 2011 to 44.9% in 2018 (2, 3). The conduct of PGT entails a biopsy of the trophectoderm layer of the blastocyst stage embryo, after which the extracted DNA is analyzed by techniques such as next-generation sequencing. Although PGT is performed with various indications in mind, including the exclusion of single gene variants or structural chromosomal rearrangements, the recent increase in the frequency of its deployment is being driven by its use to rule out aneuploidy (PGT-A) in selecting among possible embryos to implant. Importantly, for our purposes, the conduct of PGT-A also reveals the sex chromosome composition of each preimplantation embryo. Thus, in the course of PGT-A, an increasing proportion of patients who undergo IVF has an opportunity to engage in preimplantation sex selection.

Although the coupling of IVF with preimplantation sex selection has been available since the 1990s, the practice has been mostly limited to elective family balancing or, in rare cases, to the prevention of sex-linked genetic disorders. With the recent widespread use of PGT, circumstances have changed materially. Recent US national data indicate that the male-to-female sex ratio of neonates was significantly higher after IVF with PGT for any indication than without it (115 vs. 105) (4). The sex ratio of neonates proved especially pronounced after PGT for elective sex selection, with 164 males born for every 100 females (4). Although PGT was performed primarily for the purpose of elective sex selection in fewer than 7.4% of IVF cycles, the skewed neonatal sex ratios observable in the wake of *all* PGT-inclusive cycles suggest that sex selection may transpire even among patients who did not specifically seek this goal out in advance of their fertility treatment. Furthermore, the data indicate that the use of PGT is rapidly growing for both elective sex selection and other indications. Asian and White female patients appear to be using PGT at higher rates than their Hispanic and Black counterparts (4). This is likely due to socioeconomic disparities because PGT is mostly self-funded by patients even when the cost of IVF is covered, in part or in whole, by insurance.

The legal framework for the conduct of PGT for the purpose of elective sex selection varies widely from country to country. Many countries, as diverse as Chile, Australia, Austria, and Ireland, restrict the use of PGT, especially for nonmedical reasons such as elective sex selection (5). By contrast, in Saudi Arabia and Lebanon, sex selection is the most common reason to undertake PGT (6). In patriarchal societies such as India and People’s Republic of China, the strong cultural preference for male children has led to the misuse of medical technology, mostly via selective termination of female fetuses. The resulting imbalances in neonatal sex ratios, with millions of girls missing from the population, have prompted bans on all forms of sex selection (7, 8, 9, 10). Differences in the legal availability of sex selection have prompted infertile couples from around the world to travel to the United States for the conduct of IVF with PGT. Indeed, foreign nationals who undergo IVF in the United States make use of PGT and third-party reproduction, especially oocyte donation and gestational carriers, at rates far higher than those seen among US residents (11).

The Ethics Committee of the American Society for Reproductive Medicine has wrestled with the propriety of sex selection on several occasions. A 1999 committee opinion concluded that “[p]reimplantation genetic diagnosis used for sex selection to prevent the transmission of serious genetic disease [was] ethically acceptable” but that “IVF with PGT solely for sex selection… should be discouraged” (12). By contrast, the 2015 committee report softened its stance somewhat when indicating that the committee “has not reached consensus on whether it is ethical for providers to offer assisted reproductive technologies for sex selection for nonmedical purposes” and recommended instead the disclosure of practices (“clinics are encouraged to draft and make available written policies setting forth whether and under what circumstances nonmedical sex selection will be available”) and suggested that clinic employees who object to nonmedical use of PGT should be allowed to refuse to provide it (13). In 2022, the committee once again revisited the issue. In its “key points” at the start of the opinion, the committee states that “[s]ex selection should not be encouraged for nonmedical indications.” However, in the text of its conclusion, the committee seems to take a more equivocal position, noting that “assisted reproductive technologies practitioners who currently offer or decline to offer sex selection for nonmedical purposes do so against a varied ethical backdrop,” counseling full discussion of benefits and risks with patients, and once again stating that “clinics are encouraged to draft and make available written policies setting forth whether and under what circumstances nonmedical sex selection will be available” (14, 15). The Committee on Ethics of the American College of Obstetricians and Gynecologists, for its part, opposes all elective sex selection practices, on the basis that they “may ultimately support sexist practices” (16).

Elective sex selection may skew sex ratios and serve to reinforce societal oppression of girls and women. However, some view restricting or prohibiting preimplantation sex selection and postimplantation sex-selective abortion as incompatible with reproductive autonomy (8). Still, others worry that the practice may persist underground even in the face of such prohibitions (8).

In the wake of the Dobbs v Jackson Women’s Health decision rejecting a constitutional right to abortion in the United States, we are likely to see further intertwining between attempts to restrict abortion and IVF (17, 18). In the abortion context, so-called “reason bans” aimed at restricting disability and sex-selective abortion have been deployed by prolife groups to justify restrictions on abortion (19). When it comes to embryos, some fear that legal restrictions on sex selection are a more palatable foray into legal restrictions on reproductive choice in IVF. To be sure, it is possible to oppose elective sex selection through PGT without ascribing personhood to those embryos: one might view the moral evil of PGT with sex selection as stemming from the message that sex selection sends about valuing already-born children of one sex over another. One might oppose PGT with elective sex selection entirely out of concern for the sex ratio discordance that results in the children who are born. Of course, this raises the question of whether a society is morally justified in valuing a particular sex ratio; consider this biologically counterfactual thought experiment, if the prevailing sex ratio in a society was “naturally” 125:100, would we think it important to *encourage* sex selection by PGT?

But although opposing PGT for elective sex selection does not logically commit one to assigning personhood to embryos, in the current political environment, one might reasonably worry it becomes a path to exactly such a result. In the United States, regulation of IVF, PGT, and sex selection is lacking but is sure to be reconsidered in the context of the repeal of Roe v Wade (20). It is our hope that coherent regulation that protects reproductive autonomy while placing coherent limitations on elective treatments aimed at protecting patients and future generations will emerge.

## References

[1] Kushnir V.A., Smith G.D., Adashi E.Y. (2022). The future of IVF: the new normal in human reproduction. Reprod Sci.

[2] Chang J., Boulet S.L., Jeng G., Flowers L., Kissin D.M. (2016). Outcomes of in vitro fertilization with preimplantation genetic diagnosis: an analysis of the United States Assisted Reproductive Technology Surveillance Data, 2011-2012. Fertil Steril.

[3] Hipp H.S., Crawford S., Boulet S., Toner J., Sparks A.A.E., Kawwass J.F. (2022). Trends and outcomes for preimplantation genetic testing in the United States, 2014-2018. J Am Med Assoc.

[4] Bedrick B.S., Tipping A.D., Nickel K.B., Riley J.K., Jain T., Jungheim E.S. (2022). State-mandated insurance coverage and preimplantation genetic testing in the United States. Obstet Gynecol.

[5] Daar J., Cohen I.G., Mohapatra S., Suter S. (2022).

[6] Lemke T., Rüppel J. (2019). Social dimensions of preimplantation genetic diagnosis: a literature review. New Genet Soc.

[7] Purdy L. (2007). Is preconception sex selection necessarily sexist?. Reprod Biomed Online.

[8] Zilberberg J. (2007). Sex selection and restricting abortion and sex determination. Bioethics.

[9] Oomman N., Ganatra B.R. (2002). Sex selection: the systematic elimination of girls. Reprod Health Matters.

[10] George S.M. (2006). Millions of missing girls: from fetal sexing to high technology sex selection in India. Prenat Diagn.

[11] Levine A.D., Boulet S.L., Berry R.M., Jamieson D.J., Alberta-Sherer H.B., Kissin D.M. (2017). Assessing the use of assisted reproductive technology in the United States by non-United States residents. Fertil Steril.

[12] (1999). Sex selection and preimplantation genetic diagnosis. The Ethics Committee of the American Society of Reproductive Medicine. Fertil Steril.

[13] Ethics Committee of the American Society for Reproductive Medicine (2015). Use of reproductive technology for sex selection for nonmedical reasons. Fertil Steril.

[14] Ethics Committee of the American Society for Reproductive Medicine (2022). Use of reproductive technology for sex selection for nonmedical reasons: an Ethics Committee opinion. Fertil Steril.

[15] Ethics Committee of the American Society for Reproductive Medicine (2018). Disclosure of sex when incidentally revealed as part of preimplantation genetic testing (PGT): an Ethics Committee opinion. Fertil Steril.

[16] Committee on Ethics, American College of Obstetricians and Gynecologists (2007). ACOG Committee opinion no. 360: sex selection. Obstet Gynecol.

[17] Dobbs v. Jackson Women’s Health Organization. https://www.supremecourt.gov/opinions/21pdf/19-1392_6j37.pdf.

[18] Cohen I.G., Daar J., Adashi E.Y. (2022). What overturning Roe v Wade may mean for assisted reproductive technologies in the US. J Am Med Assoc.

[19] Box v Planned Parenthood of Indiana and Kentucky, Inc. https://www.supremecourt.gov/opinions/18pdf/18-483_3d9g.pdf.

[20] Feinberg E.C., Kawwass J.F., Cedars M.I. (2022). Roe v Wade and the threat to fertility care. Obstet Gynecol.

